# A Microfluidic Approach for Intracellular Delivery into Red Blood Cells: A Deeper Understanding of the Role of Chemical/Rheological Properties of the Cellular Suspension

**DOI:** 10.1007/s10439-025-03678-2

**Published:** 2025-02-19

**Authors:** Clara Bernardelli, Monica Piergiovanni, Elena Bianchi, Carmelo Carlo-Stella, Maria Laura Costantino, Giustina Casagrande

**Affiliations:** 1 Laboratory of Biological Structure Mechanics LaBS, Department of Chemistry, Materials and Chemical Engineering “Giulio Natta”, Politecnico di Milano, Milano, Italy; 2 MERYLO’ srl, Varedo, MB Italy; 3 Humanitas University, Milano, Italy

**Keywords:** Intracellular delivery, Red blood cells, Erythrocytes, Microfluidics, Encapsulation

## Abstract

Red Blood Cells (RBCs) are a promising drug delivery system candidate for many drugs. Using autologous cells helps to overcome biocompatibility issues, while microfluidics allows accurate control of the intracellular delivery of molecules through fluidic shear stress. With the ultimate goal of exploiting this delivery technique for clinical applications, we investigate how the chemical/rheological characteristics of the suspension and the properties of the RBCs in different animals influence the delivery mechanism. As regard the suspension of RBC, we study the effects induced by the hematocrit and by the presence of proteins such as albumin (Bovine Serum Albumin—BSA). Regarding the cellular properties of RBCs, we aim to investigate the exportability of the technique to the RBC of the most used animal models and identify the most suitable one. The presence of BSA implies a more significant variability of the intracellular delivery. However, 70 ÷ 94% of the cells have successfully encapsulated the probe molecule. Regarding the effect of hematocrit, however, the implementation of the experiment is more challenging due to the increase in viscosity and the easier sedimentation at low flow rates. Evaluation of intracellular delivery in the RBCs of various animal samples has instead led to the proposal of the mouse as the most suitable model for preclinical studies on this particular delivery approach.

## Introduction

Modifying the pharmacokinetics and the bio-distribution of therapeutic agents is a strategy to improve therapeutic efficacy and minimize adverse reactions. Available artificial drug carriers are based on different technologies, going from macromolecules, like micelles and liposomes that offer low systemic toxicity, to nanoparticles and peptides, which can increase the drug specificity by selective binding [1, 2]. Regulatory authorities have already approved some of these delivery systems or are undergoing clinical trials [3, 4], but several challenges still exist. First, material-related toxicity is still an issue for many nanomaterials, with concerns on inflammatory-related response, hemocompatibility, and effects on reproductive systems [5]. Moreover, carrier absorption, distribution, and metabolism are still not completely understood [6] and require considerable effort to obtain reliable products [7].

Natural carriers, such as Red Blood Cells (RBCs), can be a promising alternative for different bioactive substances to overcome some of the above-cited challenges [8–10]. RBCs have the unique advantage of being biocompatible when the patient’s blood is used and available in very high numbers by collecting a small blood sample. The absence of a nucleus makes RBCs suitable to transport a relatively high quantity of drugs. RBCs have a life cycle of around 120 days, during which they reach every district of the body. Most importantly, autologous cells can even be treated at the patient’s bedside, opening up many possibilities to revolutionize the field [11, 12].

Two main approaches to the use of RBCs as drug transporters are reported in the literature. The first consists of coupling a molecule to the cytoplasmic membrane [12], while the second is based on the loading of the compound inside the RBC (intracellular delivery) [10, 13–18]. The field of intracellular delivery of various types of molecules is of high interest for gene editing approaches, cell therapy, and basic research due to the many combinations of both cell types and cargo that can be addressed. Membrane-disruption techniques are a group of strategies to create transient pores on the cell membrane, thus allowing for the diffusion of molecules from an external, aqueous solution inside the cells. Among these, exposition to hypotonic solutions is a well-known method that is now moving to clinical application [13–16]. Other possibilities to temporarily disrupt the RBC’s membrane and obtain intracellular delivery are to use solid contact for rapid cell deformation [19] or to exploit fluidic shear stresses [17, 18, 20, 21]. Recently, the advancement in microfluidic techniques has allowed for the rapid development of these techniques, thanks to the accurate control of the fluidic shear stress conditions in the devices. In the previous works of Casagrande and Piergiovanni [17, 18], it is demonstrated that fluorescent molecules can be efficiently delivered into human RBCs upon proper fluidic shear stress conditions, while preserving their membrane integrity and physiological morphology.

Given future clinical applications, this work aims to investigate how the chemical/rheological characteristics of the suspension and the properties of the RBCs influence the intracellular delivery mechanism. A hematocrit (Ht) of up to 10% and the presence of albumin are used to replicate the physiological conditions in an experimental fluidic suspension. The assessment of the replicability of the intracellular delivery, also in RBCs derived from species other than humans, plays a key role. Since the creation of temporary porosities in the RBC membrane is strictly connected to the physiological properties of the membrane itself, it is not obvious that the process will have the same effectiveness in the presence of RBCs derived from different species. The proposed tests aim to help identify the optimal animal model for future preclinical studies.

## Materials and Methods

### Blood Supply and Species-Related RBC Characteristics

Human RBCs were separated from buffy coats from anonymous healthy human donors (nine males and three females). The buffy coats were provided to Istituto Clinico Humanitas (ICH) from authorized transfusion centers based on specific agreements, as the ICH Ethical Committee approved on January 28, 2016.

The same intracellular delivery procedures were evaluated on blood from different animal models, namely mice (*Mus musculus*—3 samples) and rats (*Rattus norvegicus*—3 samples), the most used for pharmacokinetic and prolonged drug release studies. Rat and mouse RBCs were separated from blood provided by MTTLab (Medical Transfer Trieste Laboratories, Udine, Italy) (Authorization n#546/2019-PR, 23 July 2019). Blood was withdrawn the day before the procedure and treated with Ethylenediaminetetraacetic acid (EDTA) to prevent coagulation.

The species-related different characteristics of the considered RBCs are in Table 1.

**Table 1:** Species-related characteristics of human, rat, and mouse red blood cells in terms of diameter, mean cellular volume (MCV), and hemoglobin content (Mean Corpuscular Hemoglobin, MCH), the amount of hemoglobin inside the RBC related to its dimension (MHC/MCV = MCHC or Mean Corpuscular Hemoglobin Concentration) [22], as well as in term of maximum Elongation Index (EI_max_), and shear stress required to elongate at half of EI_max_ (K_EI_) [23]

	Human	Mouse	Rat
Diameter (µm)	6–8	5.5–6	6–6.5
MCH (pg)	31	15.5 ± 3.5	22.6 ± 0.7
MCV (fL)	94	48–56	61.5–68
MCHC (%)	34.1	30.5	32.5
EImax	0.571	0.453	0.522
K_EI_	2.32	0.74	0.8

### RBCs Suspension Preparation

Whole blood was first diluted 1:3 v/v with Phosphate Buffer Saline (PBS, Sigma-Aldrich Srl, Milan, Italy) and centrifuged at 3000 rpm for 5 min (EBA 20 centrifuge, Hettich Italia Srl, Milan, Italy) to separate RBCs from plasma and other cells. For rat and murine blood, a previous centrifugation (800 rpm for 20 min) on Lympholyte (Cedarlane labs, Burlington, Canada) was necessary to accurately separate RBCs from the reticulocytes, which are present in higher percentages in rat and mouse blood compared to human blood samples [22, 23].

Bovine Serum Albumin (BSA—Sigma-Aldrich Srl, Milan, Italy) has been used to evaluate the effect of protein molecules in the solution in controlled conditions.

After the supernatant removal, RBCs derived from the same sample were re-suspended, part at 1% Ht, either in simple PBS buffer or GASP buffer (PBS with 1% (w/v) BSA [17, 24]). The second part was suspended at 10% Ht in GASP buffer to approach the possible applications, simultaneously considering the effect of the presence of albumin and the increase in hematocrit.

To test the encapsulation efficacy, coherently with literature experiments [17, 18], accounting for its good water and blood compatibility, solubility, and low toxicity, and its availability in the fluorescent-labeled form, 40 kDa Fluorescein Isothiocyanate (FITC) dextran (FD40S, Sigma-Aldrich Srl, Milan, Italy), having a Stoke radius just higher than the one of hemoglobin, was used as a probe molecule and added at 0.1 M concentration (4 mg/mL) in1% Hematocrit (Ht) RBCs solutions. Considering the number of RBCs in suspension, a 0.4 M concentration (16 mg/mL) was used with 10% Ht to guarantee sufficient probe molecules available for encapsulation. The value is chosen to maximize the number of probe molecules and limit the increase in viscosity due to the presence of dextran.

An untreated control (UT) was collected from the RBC solution before FD40S addition to monitor the auto-fluorescence of RBCs. Furthermore, a negative control (N) was collected before the intracellular delivery procedure to verify the contribution of the free diffusion of Dextran molecules through the RBC membrane.

**Fig. 1:**
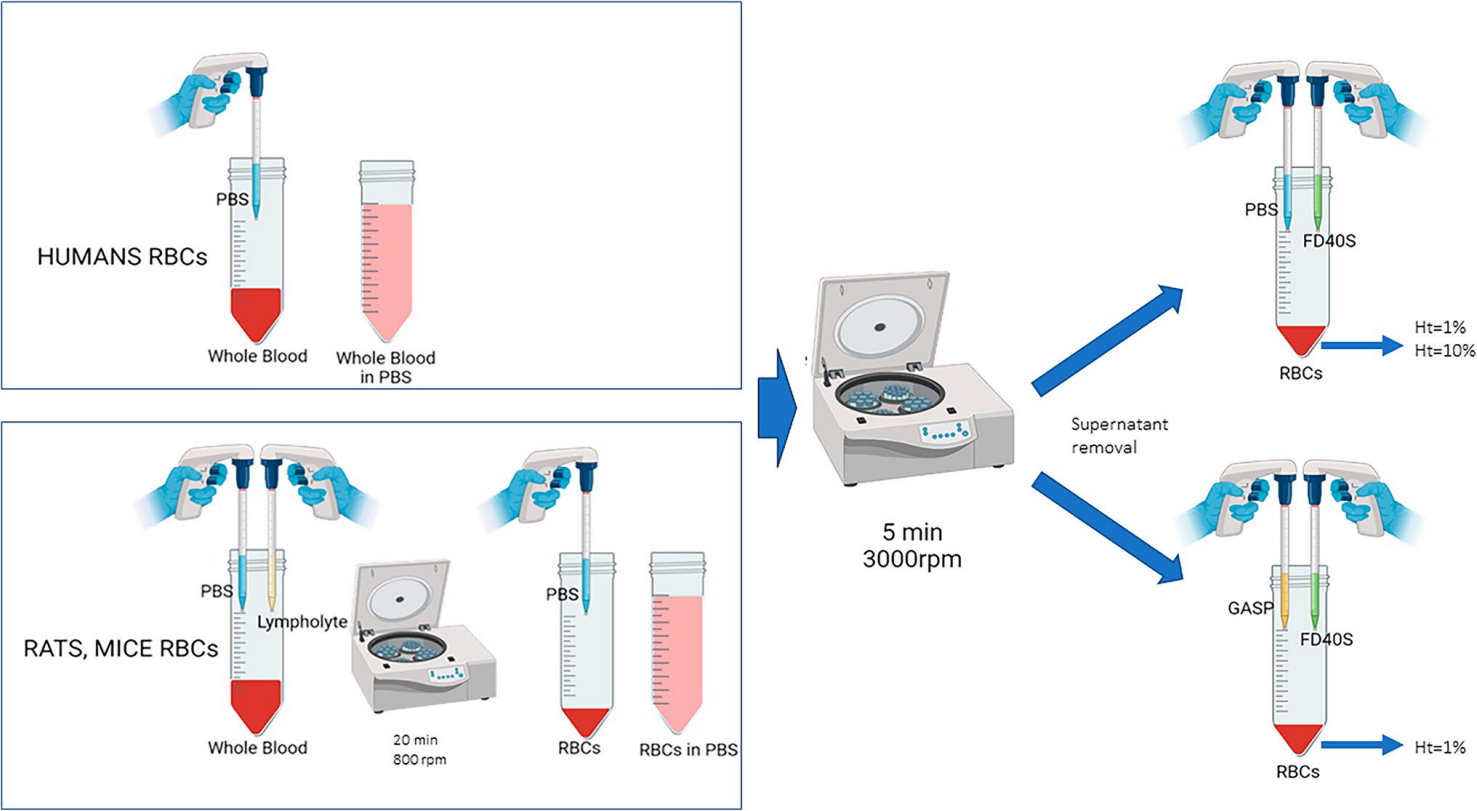
RBCs suspension preparation. Human whole blood is diluted in PBS (top left box) and prepared for centrifugation. For rats and mice blood, a preliminary density gradient centrifugation with Lympholite is performed (bottom left box). After the supernatant removal, the rat’s and mice’s RBCs were diluted in PBS. Both the diluted samples were centrifuged at 3000 rpm for 5 minutes. After the supernatant removal, RBCs derived from the same sample were re-suspended, part in simple PBS buffer (top right) and part in GASP buffer (PBS with 1% (w/v) BSA—bottom right). FD40s dextran was added as a probe molecule in both. The suspension was prepared either at 1 or 10% Ht for human blood, only at 1% for rats’ and mice’s blood. “Created in BioRender. Bernardelli, C. (2024) https://BioRender.com/z79b573”

The same protocol, as resumed in Fig. 1, was performed on rat and mouse samples with the only difference in the initial procedure for separating the RBCs, as previously described.

### Protocol for Intracellular Delivery

A poly-methyl-methacrylate chip (Microfluidic ChipShop GnbH, Jena, Germany; Product code #10001814) with a nominal square section of 50 μm and a length of 58.5 mm was used as a microfluidic device for intracellular delivery. A syringe pump (Harvard Apparatus PHD 2000, Massachusetts, USA) equipped with a 1 mL glass syringe (Hamilton Gastight Syringes, Nevada, USA) pushed the RBCs suspension through a 25 G needle and a Polytetrafluoroethylene (PTFE) tube (ID 0,5 mm; OD 1,6 mm; Bola, Bohlender GmbH, Grünsfeld, Germany), connected to the chip with an ad hoc mini Luer port (Microfluidic ChipShop GmbH, Jena, Germany).

**Fig. 2:**
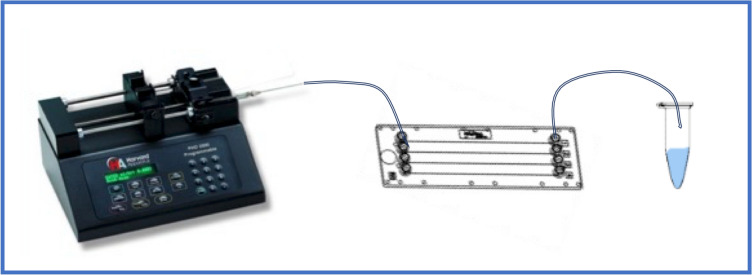
Schematic of the experimental setup. Pump, microfluidic chip, and reservoir. The chip includes four independent channels, each one with is Mini Luer port

The use of the syringe pump allowed the use of a fixed, that was stepwise increased (5–30–50 µL/min) to find the best match of fluid dynamic solicitation imposed on RBCs membrane and solicitation time, useful to obtain the maximum encapsulation efficacy, minimizing the membrane damages [17], and sedimentation. The scheme of the experimental setup is in Fig. 2, and the summary of the performed tests is in Table 2.

**Table 2:** Summary of the different tests performed

RBCs	Solvent	[FD40s]–Ht%	#tests	Flow rates [µL/min]
*Human*	PBS	0.1 M–1%	3	5 − 30 − 50
	GASP	0.1 M–1%	3	5 − 30 − 50
	GASP	0.4 M–10%	4	5 − 30 − 50
*Rat*	PBS	0.1 M–1%	2	5 − 30 − 50
	GASP	0.1 M–1%	2	5 − 30 − 50
*Mice*	PBS	0.1 M–1%	3	5 − 30 − 50
	GASP	0.1 M–1%	3	5 − 30 − 50

### Flow Cytometer Analyses

Collected samples (150 µL each) were rinsed (three times) in PBS to remove excess dextran. RBCs were then analyzed by using a flow cytometer (BD FACS Canto II, BD Biosciences, Becton Dickinson Italia, Milan, Italy) to measure the increase of fluorescence, proportional to the efficacy of FD40S encapsulation, compared to the N and UT samples. The signal of Side Scatter (SSC) and Forward Scatter (FSC) in Untreated samples (UT) was used to select the threshold for the analysis of the RBC population whose morphology was not substantially altered and to eliminate debris that could derive from membrane disruption. In particular, normal RBCs, with the physiological biconcave shape, were distinguished from altered ones, namely echinocytes or stomatocytes. Each measure was taken twice on a 20,000-cell sample.

The fluorescence was evaluated by taking the geometrical mean of the sample ($\overline{F }$), calculated with Eq. (1), where *x*_*i*_ is the fluorescence of each cell and *n* is the total number of cells.

1 $$\overline{F }=\frac{log\sum {x}_{i}}{n}$$

The intracellular delivery efficiency is influenced by two parameters: the quantity of dextran delivered to cells and the number of cells that receive at least one molecule. The first parameter is directly related to the observed fluorescence and was calculated with Eq. (2) by comparing the fluorescence of the sample and the one of N control (free diffusion of the probe molecule).

2 $$\Delta F[\%] = \frac{{\overline{F} }^{sample}- {\overline{F} }^{control}}{{\overline{F} }^{sample}}$$

The number of Loaded Cells (LC) cells is calculated as the percentage of cells whose fluorescence increased after treatment. This parameter can be automatically calculated by the BD DIVA Software after setting the reference condition (N control).

### Statistical Analyses

Each group of results was described in terms of average and standard deviation. Statistically significant differences in the parameter’s values have been evaluated using *t* tests, considering a *p* value threshold of 0.05.

## Results

### Intracellular Delivery in Human RBCs at 1% Ht in the Presence of BSA Protein

**Fig. 3:**
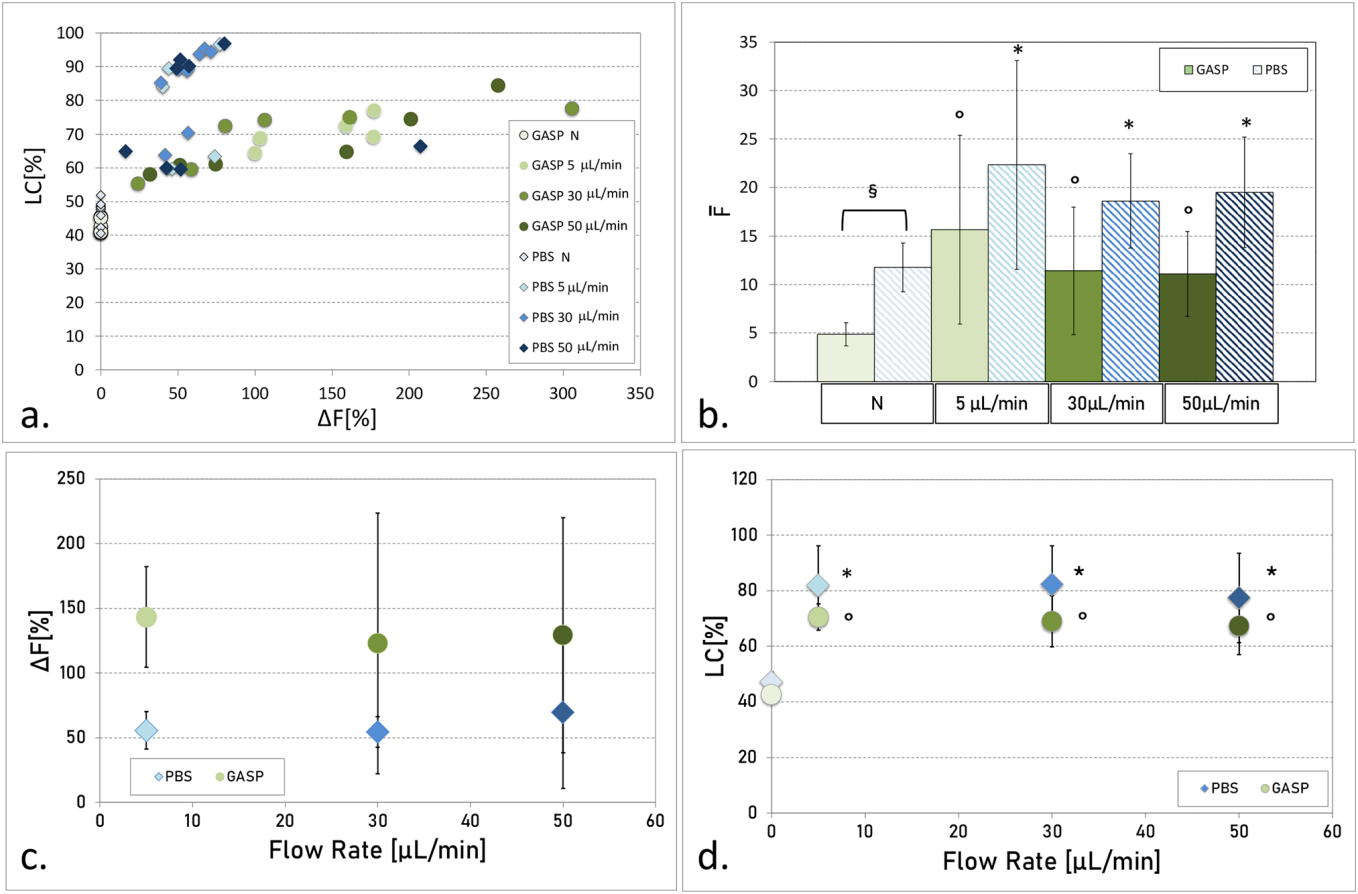
Flow cytometry results for the encapsulation tests in PBS (blue tones) and GASP (green tones) solutions. The color code is maintained for the four panels. **a.** LC vs ΔF. **b.** The histogram compares the $\overline{\text{F} }$ (fluorescence unit after calibration with beads) in PBS and GASP, for each flow rate, 3 tests. § refers to differences between GASP and PBS at the same flow rate; ° and * to statistical differences to the N control for GASP and PBS, respectively. **c.** Treated samples and N control were compared in terms of ΔF in PBS and GASP, 3 tests. **d.** Treated samples and N control were compared in terms of LC in PBS and GASP, 3 tests. ° and * to statistical differences to the N control for GASP and PBS, respectively

All experiments were performed in triplicate on human blood. The test showed good intracellular delivery, both in terms of the quantity of delivered probe molecules and the number of cells that encapsulated the molecule. Figure 3a shows the encapsulation performance (number of loaded cells LC vs %variation in fluorescence $\Delta F$) in PBS and GASP at varying flow rates. Up to 85% of the cells encapsulated the fluorescent molecule when processed in GASP suspension, while this percentage reaches 97% in PBS. The number of loaded cells is stable at varying flow rates. The ΔF is generally lower than 100% for PBS, whereas for GASP, more distributed values are obtained. Significantly lower free diffusion of FD40S in GASP (4.88 ± 1.18) than in PBS (11.74 ± 2.51) is measured in the N control (p < 0.05) Fig. 3b). The flow rate value seems to have only a limited influence on the fluorescence of the treated samples, but comparing treated and N control (0 mL/min) samples, statistically significant differences are highlighted for all parameters considered (Fig. 3b–d).

### Intracellular Delivery in Human RBCs at 10% Ht in the Presence of BSA Protein

**Fig. 4:**
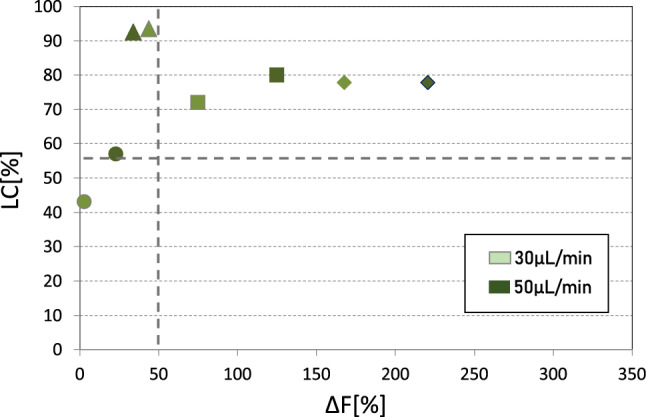
Results of the encapsulation test for FD40S considering solutions 0,4 mM at 10% Ht, in GASP. A different marker shape refers to the four experiments. Lighter green refers to the 30 µL/min flow rate, while intense green is 50 µL/min. Dotted lines refer to the minimum values to consider a significant increase of LC and ∆*F* [17]

Four experiments of intracellular delivery on human RBCs at 10% Ht in GASP have been performed. The probe molecule, FD40S, was used at a concentration of 0,4 mM (16 mg/mL). A high percentage of cells successfully encapsulated the probe molecule (70 ÷ 94%) except in one experiment, in which LC ranged from 40 to 60% (Fig. 4). Instead, the value of ΔF appears extremely dependent on the patient blood sample. Results that refer to the flow rate of 5 μL/min are not reported since such a low flow rate, at 10% Ht, implied high sedimentation of the cells, clogging the microchannels.

### Intracellular Delivery in Rat and Murine RBCs

**Fig. 5:**
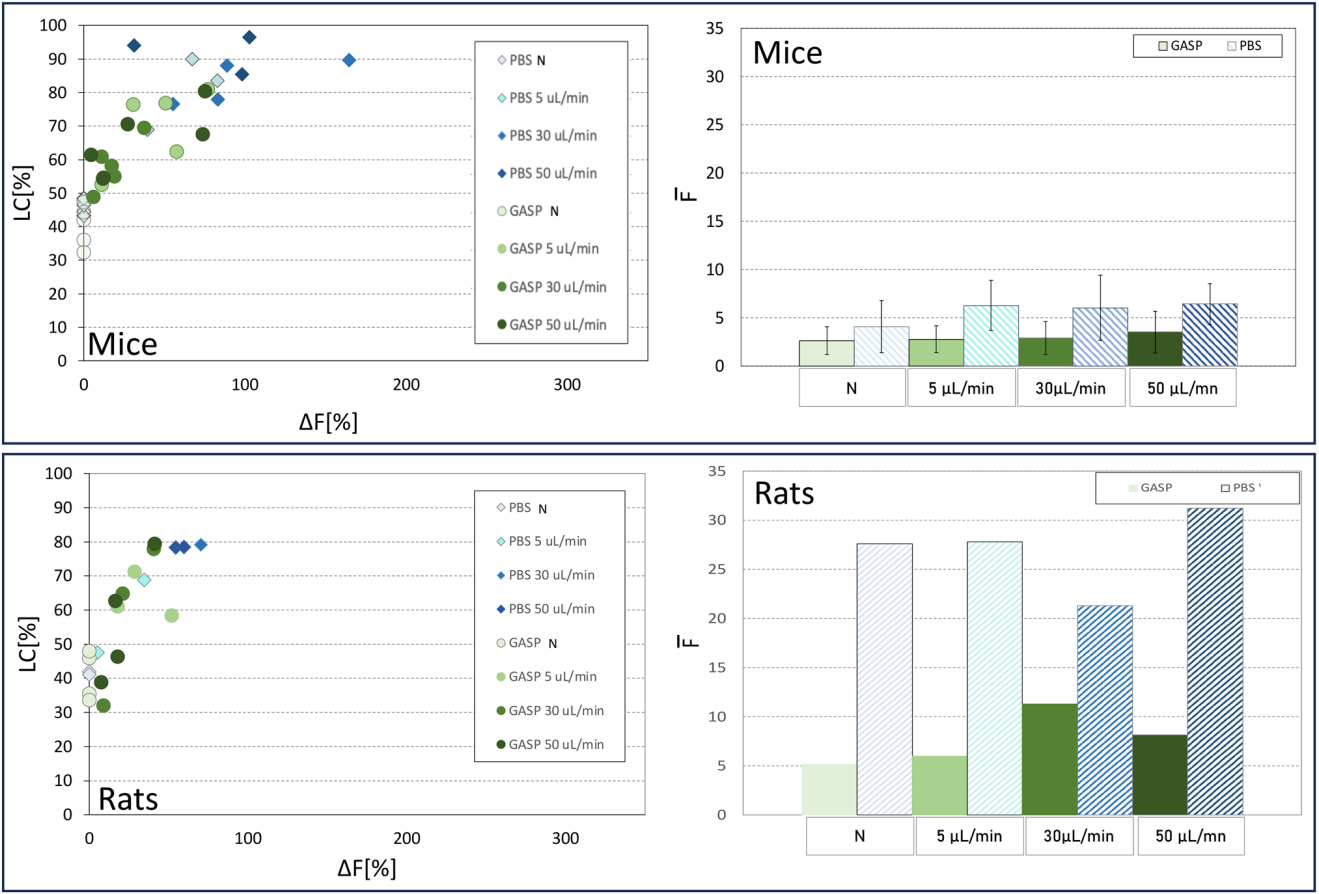
Flow cytometry results of encapsulation tests in PBS (blue tones) and GASP (green tones) solutions for mice (top, 3 tests) and rats (bottom, 2 tests). The color code is maintained along the panels. Left panels: LC vs ΔF. Right panels: The histogram allows the comparison of the average measured $\overline{\text{F} }$ in PBS and GASP for each flow rate

All the tests on mouse (*Mus musculus*) and rat (*Rattus norvegicus*) RBCs were performed at 1% Ht, using FD40S as a probe molecule. FD40S was successfully delivered to the RBCs of both animals. One test in rats failed due to partial hemolysis during the sample preparation, so the presented results only refer to two samples. The encapsulation is more effective in mice blood, with values of ΔF up to 80% in GASP (Fig. 5). Rat RBCs showed a very low encapsulation, with a maximum fluorescence increase near 50%. As already observed for human RBCs, the free diffusion of molecules is lower when RBCs are suspended in GASP rather than PBS.

## Discussion

This paper aims to investigate how the chemical/rheological characteristics of the suspension and the mechanical properties of the cells influence the intracellular delivery of molecules into RBCs by fluidic shear stresses in microchannels.

The correct evaluation of the solicitation condition considers that different hematocrits and the presence of BSA imply alterations in the viscosity of the suspension.

According to the work of Chien, at 20 °C, PBS viscosity is 1050·10^−3^ Pa·s, with BSA at 1 mg/mL the viscosity increases to 1054·10^−3^ Pa·s. Ht has a higher influence on the viscosity, and at 10% Ht, in the GASP buffer suspension, the viscosity is 1318·10^−3^ Pa·s [25].

Dextran greatly impacts the viscosity of the suspension, growing the viscosity according to the quadratic Huggins’s semi-empirical equation, Eq. (3) [26].

3 $${\mu }_{susp}={\mu }_{solv}\cdot \left[1+C\cdot \left[\mu \right]+k{\cdot \left[\mu \right]}^{2}\cdot {C}^{2}\right]$$

where C is the polymer concentration, k is an experimentally determined parameter (for Dextran in PBS or GASP equal to 0.3—flexible chains in good solvents [26]), and [*µ*] the intrinsic viscosity of FD40S. The intrinsic viscosity is determined with the Mark–Houwink formula, Eq. (4) [27]:

4 $$\left[\mu \right]=K{{\cdot M}^{\alpha }}$$

where M is the molecular mass of the polymer, K and *α* are two constants depending on the solvent (K = 0.1361*10^−3^ and *α* = 0.45 for PBS [28]). The viscosities of the suspensions used for the experimental tests as well as of the intermediate conditions implying the variation of a single factor at a time, estimated according to the cited equations, are reported in Table 3.

**Table 3:** Viscosities and diffusivities of the suspensions used for the experimental tests (*) and of intermediate conditions, estimated according to the equations presented by Chien, Pamies, Yan, and Masuelli [25–28 ]

Ht [%]	Solvent	Dextran—M [kDa]	Intrinsic Viscosity FD40—µ [Pa·s]	C_dextran [g/mL]	µ_susp [Pa·s]	Diffusion coefficient—D [m^2^/s]
1	PBS*	40	16.025·10^−3^	0.004	1.1186·10^−3^	1.46·10^−12^
1	GASP*	40	16.025·10^−3^	0.004	1.1229·10^−3^	1.45·10^−12^
1	GASP	40	16.025·10^−3^	0.016	1.450·10^−3^	1.21·10^−12^
10	GASP	40	16.025·10^−3^	0.004	1.4041·10^−3^	1.16·10^−12^
10	GASP*	40	16.025·10^−3^	0.016	1.6819·10^−3^	9.68·10^−12^

Experimental tests with BSA showed lower fluorescence than the measures in simple PBS, regardless of the source of the RBCs. When measuring the free diffusion of dextran, a decrease of $\overline{F }$ (on average − 33% for human RBCs, − 55% for mice RBCs, and − 81% for rat’s RBCs) was always detected in the N control when BSA was added. This large difference cannot be explained by the slowed diffusivity due to the increase in viscosity (viscosity + 0.32% and diffusivity − 0.38% in GASP vs PBS at 1% Ht), but rather by a molecular interaction between BSA and dextran. Dextran molecules can form inter-polymeric complexes with BSA, either at low or high temperatures [29]. This phenomenon is amplified when excess polysaccharides are present in the solution and if the protein exists in its native state. In particular, an excess of dextran induces significant structural changes. The main variations occur at the level of the secondary and tertiary structures. This interaction is likely due to the formation of hydrogen bonds between the hydroxyl groups of the polysaccharide and the tryptophan residues located on the surface of the protein globule [29, 30].

Despite that, the application of controlled stresses in the microfluidic channel (treated cells) also allows for optimal intracellular delivery in the presence of BSA. Even if the number of cells involved is slightly lower than with PBS (~69% in GASP, ~ 80% in PBS), more dextran can be entrapped into RBCs if the GASP solution is used (+71%), increasing the efficiency of intracellular delivery.

Consequently, the presence of proteins in suspension favors the formation of complexes that mainly limit the free diffusion but do not impede the loading of the probe molecules. It can be supposed that the fluidic shear stress breaks down part of the bonds between BSA and dextran, and the temporary pores on the RBC membrane allow diffusion of the cargo molecule. Then, being GASP a more accurate representation of whole blood, it is chosen as the preferential solvent for the next tests.

When increasing the Ht to 10%, the efficiency of the intracellular delivery is substantially comparable to results at 1% Ht (Figs. 3 and 4—stable average LC%, − 1%; slightly decreased average ΔF%, − 6%).

The slight decrease in ΔF can be explained considering that the proposed increase in concentration ([FD40S] = 0.4 M) and Ht (Ht = 10%) implies a viscosity increase of by 50% and a diffusivity decrease (− 33%), compared to Ht = 1% and [FD40S] = 0.1 M (Table 3).

The main role is played by Ht, whose increase from 1 to 10% causes a 25% increase in the viscosity of the solution and, therefore, a reduction in the diffusivity (− 20 %) of the dextran molecules. The increase in dextran concentration to 0.4 M alone would have implied, however, a 20% increase in viscosity and a 17% reduction in the diffusion coefficient (Table 3).

When comparing intracellular delivery at similar flow rates, it must also be considered that a higher viscosity increases the fluidic shear stresses acting on the flowing cells. Considering the laminar condition of the considered flows, the maximum shear stress in the microchannel is calculated as the product between shear rate and viscosity, and with 1% Ht is 49.7 Pa, the flow rate is 50 μL/min, but the same maximum shear stress can be obtained with a flow rate of only 28 μL/min for the solution with 10% Ht.

The fact that the flow rate value seems to have only a limited influence on the fluorescence of the treated samples (Fig. 3), as long as the conditions remain sub-hemolytic, could be justified by the fact that the RBCs tend to arrange themselves in a circular coronal region, leaving close to the walls just a layer of plasma [31]. The variations in the shear stress experienced by the cells are, therefore, lower than those observed if they were estimated at the wall where the shear is maximum.

The process is sub-hemolytic (we do not exceed the threshold of membrane integrity), as highlighted by the flow cytometry, light microscopy, and confocal microscopy analyses in previous works, demonstrating the preservation of the RBC’s characteristic biconcave morphology with a size comparable with N controls [17, 21]. Flow cytometry confirms this result also in the present study.

When using RBCs from different animal models, a lower encapsulation efficacy than with human RBCs is generally observed. The intracellular delivery, in terms of ΔF, in PBS, in rats and mice, appears decreased vs. human RBCs (around − 82% and − 73%, respectively). This difference might be ascribed to the physical properties of the mouse and rat RBC membranes.

The literature describes a direct correlation between deformability and MCV values of various species [32], suggesting that RBCs with a smaller volume do not have to deform as much to pass through the microcirculation compared to larger RBCs. Correlations with cell volume show that smaller cells are more fragile in hypotonic and more resistant to hypertonic media [33]. Moreover, the structural composition of the cell membrane is different between humans, mice, and rats. The lack of specific phospholipids in mice and rats increases their osmotic fragility.

Specifically, rats and mice RBCs are slightly smaller (− 11 and − 17% in diameter, respectively; − 32 and − 45% in volume, respectively) [23] and less deformable (Elongation index, EI_max_, decrease equal to − 20 and − 8,6%, respectively; K_EI_ decrease equal to − 66 and − 68%, respectively) [32]. Lower EI_max_ and especially K_EI_ values ​​confirm that rats’ and mice’s RBCs are less deformable than human RBCs. These mechanical properties will influence the choice of the flow rate values to be tested since the parameters suitable for human blood may not be effective for rats and mice RBCs.

Finally, the differences in RBC aggregation that exist among the considered species, both for cells suspended in autologous plasma and polymer solutions, can justify the observed results. Mouse and rat RBCs exhibit, in fact, very low or no aggregation in plasma or polymers [34, 35], differently from human RBCs.

To identify an adequate animal model for future in vivo studies, we expect a model in which the number of loaded cells LC is similar to that of human RBCs because the number of cells available to be treated is smaller in these models. We can instead accept a decrease in loading efficacy, possibly evaluating different choices of drug concentration in the suspension to maintain therapeutic efficacy. These considerations point out the mouse as the most suitable animal model.

Summarizing, intracellular delivery in different autologous cells is becoming an interesting drug delivery technique in many fields. When coming to RBCs, their unique membrane properties require a specific combination of applied force and time to avoid hemolysis. These conditions can be met and controlled by using microfluidics, as shown in the previous work. Using BSA to reproduce the plasma protein content and a solution with 10% hematocrit led to the successful delivery of the probe molecule in 90% of the treated human cells. Using rat and mouse blood allowed us to account for the different mechanical properties of RBCs that can influence intracellular delivery efficiency. Mouse, at this preliminary stage, seems the most stable and suitable model for future preclinical safety tests.
